# Questionable Validity of Creatinine-Based eGFR in Elderly Patients but Cystatin C Is Helpful in First-Line Diagnostics

**DOI:** 10.3390/geriatrics8060120

**Published:** 2023-12-08

**Authors:** Dario Geißer, Lina Hetzel, Ralf Westenfeld, Fritz Boege

**Affiliations:** 1 Central Institute of Clinical Chemistry and Laboratory Diagnostics, Medical Faculty, Heinrich Heine University and University Hospital, 40225 Düsseldorf, Germany; boege@med.uni-duesseldorf.de; 2 Division of Cardiology, Pulmonology and Vascular Medicine, Medical Faculty, Heinrich-Heine University, 40225 Düsseldorf, Germany; lina.hetzel@hhu.de (L.H.); ralf.westenfeld@med.uni-duesseldorf.de (R.W.)

**Keywords:** renal dysfunction, chronic kidney disease, CKD stage, eGFR, creatinine, cystatin C

## Abstract

Background: The recommended chronic kidney disease (CKD) first-line diagnostic test is based on the creatinine-derived (estimated) glomerular filtration rate (eGFR). Cystatin C use may provide a better assessment. Methods: We compared creatinine- and cystatin C-derived eGFR determination as the first-line diagnostic test for 112 hospital patients aged > 60 years (median = 76 years). The patients were judged to not have CKD (no-CKD group) according to the first-line diagnostic recommendations (*n* = 61, eGFR (CKD Epidemiology Collaboration (CKD-EPI)) ≥ 60 mL/min/1.73 m^2^, total urine protein < 150 mg/g creatinine, urinary red/white blood cells not increased) or classified to be at risk for kidney insufficiency due to aortic valve dysfunction (at-risk group; *n* = 51). The accuracy of the eGFR values was evaluated retrospectively with the final case diagnoses. Results: The eGFR (Caucasian, Asian, pediatric, and adult formula (CAPA)) was found to be linearly correlated to the eGFR (CKD-EPI) (R^2^ = 0.5, slope = 0.69, *p* < 0.0001). In 93/112 (>80%) cases, the eGFR (CAPA) yielded lower values (on average ≈−20%). In 55/112 (49%) cases, the cystatin C-derived CKD stage was lower. CKD reclassification from no-CKD to a kidney-insufficient state (i.e., CKD1/2 to CKD3a/b or 4) or reclassification to a more severe kidney insufficiency (i.e., CKD3a → 3b/4 or 3b → 4) was found in 41/112 (37%) cases. A worse CKD classification (no-CKD → kidney-insufficient) based on the eGFR (CAPA) was plausible in 30% of cases in light of the final case diagnoses. Conclusion: In elderly patients (>60 years), renal function appears to be systematically overestimated by the creatinine-based eGFR (CKD-EPI), indicating that, for this group, the cystatin C-based eGFR (CAPA) should be used as the first-line diagnostic test.

## 1. Introduction

Impaired kidney function and/or chronic kidney disease (CKD) is a major cause of morbidity and mortality, particularly in societies undergoing demographic changes. Currently, the worldwide prevalence of CKD is estimated at 8–16% [1] From 1960 to 2016, death due to CKD increased by 98% to ~1.2 million per year in 2016 [2].

Detecting chronic kidney disease with first-line diagnostic tests is of significant importance. It is a comorbidity and a severe risk factor for many diseases and influences the outcome after cardiological interventions [3,4,5,6]. It must be taken into account when determining the dosage of potentially nephrotoxic medications and the application of potentially kidney-damaging radiological procedures [7].

This is true for critically ill and elderly patients with known kidney disease or at high risk of such a disease and for supposedly healthy patients with no known kidney disease. Two subaspects are also relevant here: unknown kidney damage must be reliably detected, and the stage of kidney damage must be correctly assessed.

We aimed to determine whether the guidelines for detecting chronic kidney disease are suitable for the first-line detection and classification of chronic kidney diseases in elderly patients (aged > 60 years) admitted to a hospital.

To date, noninvasive assessments of renal function and dysfunction rely mostly on laboratory tests. The clinical stages of CKD as defined by the organization “Kidney Disease: Improving Global Outcomes (KDIGO)” are entirely based on glomerular filtration rate (GFR) values [8]. The most reliable procedure for determining GFR is the plasma clearance or urinary excretion of exogenous filtration markers such as inulin or iohexol [9,10]. However, these procedures are of little use in routine clinical healthcare. Measuring the clearance of endogenous filtration markers, such as creatinine, is an alternative approach to GFR determination. This approach is more practical in the clinical context. However, the determination of endogenous creatinine clearance is not widely used because it requires the stringent collection of complete urine excretion over extended time periods. This procedure is cumbersome and impractical in most clinical situations. Instead, estimating GFR based on the plasma levels of endogenous filtration markers has become the procedure of choice.

Currently, the initial screening of renal function is almost always based on GFR estimates derived from serum creatinine using the Chronic Kidney Disease Epidemiology Collaboration (CKD-EPI) formula as recommended by international guidelines [8]. Various formulae for calculating the estimated GFR (eGFR) have been established in routine clinical diagnostics [11]. The CKD-EPI formula using serum creatinine is confounded by tubular secretion, muscle mass, food protein intake, and medications [7]. Due to these confounders, serum creatinine does not increase until GFR drops by more than 50% [12]. This limitation is crucial because a decrease in GFR of more than 50% (i.e., below 60 mL/min/1.73 m^2^, entailing a transition from CKD stage 2 to CKD stage 3) is associated with a significantly increased mortality [13]. This shortcoming has prompted the search for more reliable filtration markers, such as the cysteine protease inhibitor cystatin C [14]. But GFR estimates derived from serum cystatin C are rarely used and not recommended as a primary diagnostic test [8] despite several studies indicating the superiority of cystatin C as a marker for estimating GFR and predicting kidney function [6,14,15,16,17].

Consequently, here, we address two main questions:

1. Are kidney disease patients correctly identified using the recommended first-line diagnostic test?

For this purpose, we studied patients admitted to the hospital with various diagnoses. These patients were determined to not have CKD according to the currently recommended first-line kidney diagnostic test (eGFR CKD-EPI ≥ 60 mL/min/1.73 m^2^ and negative results of urine analysis; no-CKD group). We rechecked their renal status using cystatin C-based eGFR and used the coded diagnoses to judge the validity of the two procedures. Here, we tested the CAPA formula, the most widely implemented formula in routine healthcare.

2. Are patients diagnosed using the recommended first-line diagnostic test assigned to the correct GFR stage?

For this purpose, we additionally studied patients considered prima facie at risk of chronic kidney failure due to suffering from aortic valve stenosis and therefore, being considered for transcatheter aortic valve implantation (TAVI). Patients undergoing TAVI can be expected to have a higher incidence of CKD than the healthy population [18]. When looking at these patients, we addressed the differences in the classification of kidney dysfunction manifestations according to cystatin C- and creatinine-based GFR estimations, as previously described by others [6].

The recommended strategy may be particularly unsuitable for elderly individuals, most notably for elderly hospitalized patients. These patients are afflicted by co-morbidities compromising kidney dysfunction. They are often lined up for potentially nephrotoxic iatrogenic interventions. Furthermore, they are typically subjected to age-related decline in muscle mass and residual GFR capacity, which compromises the utility of creatinine-based GFR estimation.

## 2. Materials and Methods

Study participants: A total of 112 in- and out-patients of the University Hospital of the Heinrich Heine University Düsseldorf were included and assigned to two cohorts. One cohort (*n* = 61, 25 females, 36 males, mean age 71.89 years, median age 70.87 years) was considered no-CKD because these patients appeared to be in good renal health after the primary diagnostics that are frequently used in clinical practice according to the guidelines. These patients were included based on the following criteria: age > 60 years, eGFR CKD-EPI ≥ 60 mL/min/1.73 m^2^, total protein in urine < 150 mg/g creatinine or <150 mg/L, white and red blood cell count in urine <25/μL and <23/μL, respectively, or corresponding negative results for white and red blood cells in a urine test strip analysis. The true state of renal or nonrenal diseases of these patients was determined ex post according to the etiology of the “*International Statistical Classification of Diseases and Related Health Problems*” (ICD-10)-encoded diagnoses (see Supplementary Table S1). We decided on this type of patient inclusion because it is precisely these laboratory parameters that are used to assess renal function in the primary diagnosis of patients without known CKD.

The other cohort (*n* = 51, 23 females, 28 males, mean age 81.48 years, median age of 82.69 years) was denominated at risk (TAVI) due to suffering from aortic valve stenosis known to increase the risk of kidney dysfunction [18]. These patients were admitted to the hospital for transcatheter aortic valve implantation (TAVI) and were included in the study if aged > 60 years.

Ethics: The investigation was approved by the local ethics board of the Medical Faculty of the Heinrich Heine University Düsseldorf (study number 2022-1839). Laboratory results and ICD-10-coded diagnoses retrieved for this study were anonymized before the data analysis. The investigation conformed to the principles outlined by the Declaration of Helsinki of the World’s Medical Association. All patients gave informed consent.

Laboratory analyses: All analyses were performed by accredited standardized diagnostic procedures as part of the routine diagnostic workup of the participants. Lithium-heparin plasma or serum was obtained via vein puncture. Samples were analyzed within 2 h or stored for reflex testing at −20 °C for no more than eight weeks. Creatinine, cystatin C, and urea were determined on a Cobas 8000 analyzer (Roche; Basel, Switzerland). Creatinine was analyzed using an enzymatic assay. Cystatin C was analyzed with a particle-enhanced immunological turbidity assay. Urea was analyzed by using the coupled enzyme system urease/GLDH.

Traceability: The method of creatinine determination was standardized against ID/MS (isotope dilution mass spectrometry) according to the manufacturer’s instructions. The method of cystatin determination was standardized against the ERM-DA471/IFCC [19] reference material according to the manufacturer’s instructions. The method of urea determination was standardized against the SRM 909b [20] reference material according to the manufacturer’s instructions.

### Calculation of the eGFR

Creatinine-based GFR estimates were calculated according to the CKD-EPI formula [21] for females, plasma creatinine ≤ 0.7 mg/dL:

GFR_CKD-EPI_ = 144 × (Creatinine [mg/dL]/0.7)^−0.329^ × 0.993^age^

For females, plasma creatinine > 0.7 mg/dL:

GFR_CKD-EPI_ = 144 × (Creatinine [mg/dL]/0.7)^−1.209^ × 0.993^age^

For males, plasma creatinine ≤ 0.9 mg/dL:

GFR_CKD-EPI_ = 141 × (Creatinine [mg/dL]/0.9)^−0.411^ × 0.993^age^

For males, plasma creatinine > 0.9 mg/dL:

GFR_CKD-EPI_ = 141 × (Creatinine [mg/dL]/0.9)^−1.209^ × 0.993^age^

Creatinine-based GFR estimates were calculated according to the MDRD formula [21] as follows:

GFR_MDRD_ = 175 × Creatinine [mg/dL]^−1.154^ × age^−0.203^ × 1.212 [if black] × 0.742 [if female]

Cystatin C-based GFR estimates were calculated according to the CAPA formula [22]:

GFR_CAPA_ = 130 × Cystatin C [mg/L]^−1.069^ × age^−0.117^ − 7

Statistics: Graph Pad Prism 9 (Graph Pad Software, Inc., San Diego, CA, USA: released in 2020. Graph Pad Prism 9 for Mac, San Diego, CA, USA: Graph Pad Inc.) and Microsoft Excel (Microsoft, Redmond, WA, USA: released in 2022. Microsoft Excel for Mac Version 16.67, Redmond, WA, USA: Microsoft Corp.) were used for the analysis. The data were descriptively analyzed with mean and median values. The normality of distributions was assessed according to the Shapiro–Wilk test. Correlations were analyzed via Spearman’s correlation and assumed to be good at r ≥ 0.7 and moderate at r ≥ 0.5. Differences were analyzed with the Wilcoxon test for paired samples. For all tests, statistical significance was assumed at *p* < 0.05.

## 3. Results

### 3.1. Cystatin C-Based eGFR (CAPA) Is Linearly Correlated with but Almost Always Lower Than Creatinine-Based eGFR (CKD-EPI)

Across the entire population of 112 elderly patients, a systematic downward bias of the cystatin C-based eGFR (CAPA) relative to the creatinine-derived eGFR (CKD-EPI) was indicated by a linear regression of the two datasets, yielding

**Figure 1:**
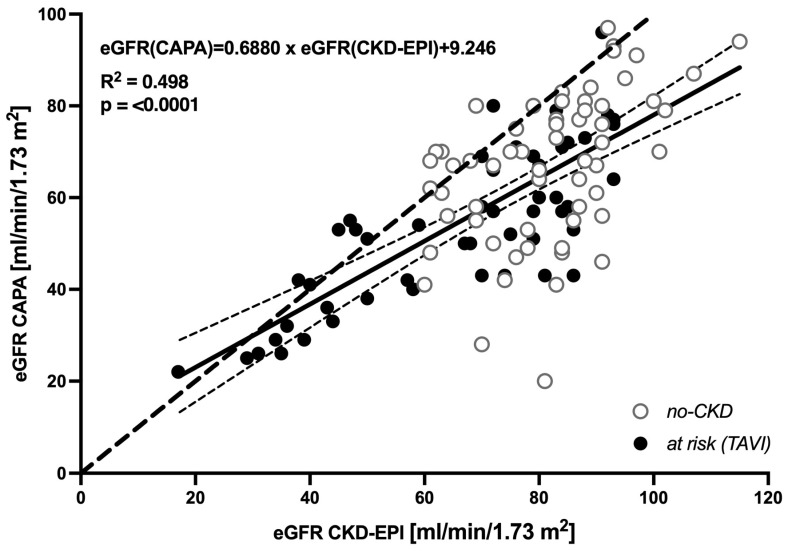
Linear regression of eGFR CKD-EPI and eGFR CAPA: Cumulated results obtained for no-CKD (*n* = 61, open circles) and at-risk (TAVI) cases (*n* = 51, closed circles). Each data point represents one patient. Solid line: linear regression of the values. Thin dotted lines: 95% confidence intervals of the linear regression. Thick dotted line: Hypothetical identity of results.

eGFR(CAPA) = 0.6880 × eGFR(CKD-EPI) + 9.246

(R^2^ = 0.498, *p* < 0.0001) (Figure 1). Notably, at eGFR > 40 mL/min/1.73 m^2^, the regression line and the 95% confidence interval of regression were far beneath the hypothetical line of identity, supporting the notion that moderate decreases in GFR are indicated by the cystatin C-based eGFR (CAPA) but not by the creatinine-based eGFR (CKD-EPI).

**Figure 2:**
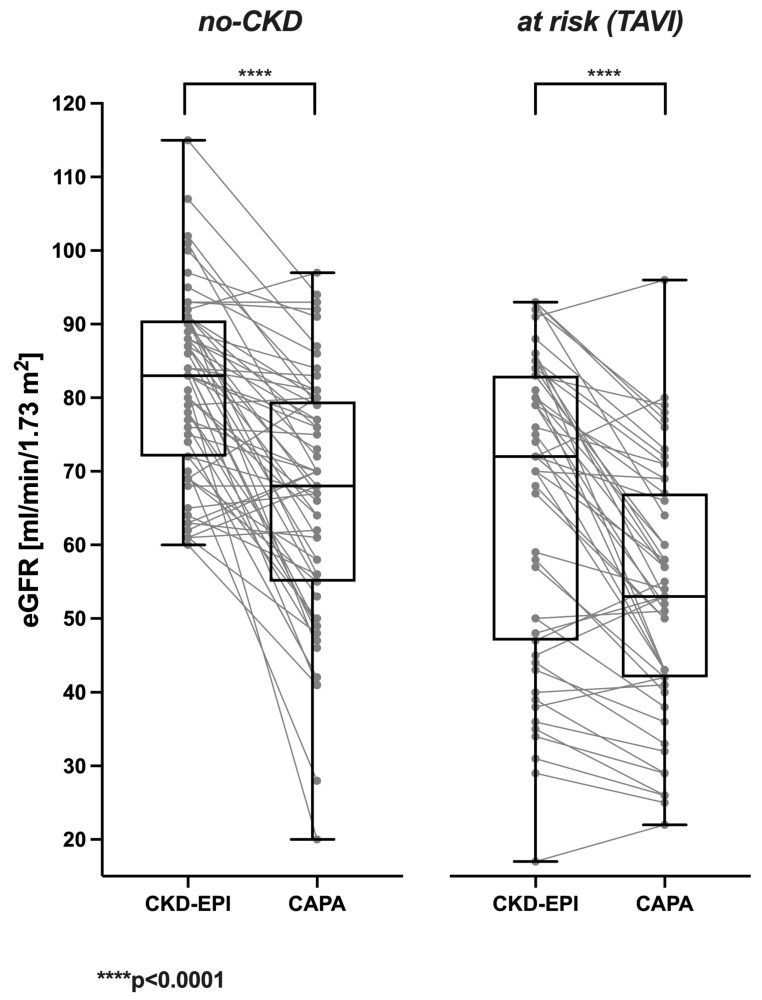
Individual differences between eGFR CKD-EPI and eGFR CAPA: Results obtained for the no-CKD (**left**) and at-risk (TAVI) (**right**) groups. Each data point represents one patient. Corresponding eGFR results obtained via CKD-EPI and CAPA are joined by lines. The box plots show quartiles. The horizontal line represents the median. The vertical line with upper and lower whiskers represents the range of values.

Correspondingly, in a vast majority of the analyzed cases, the cystatin C-based eGFR (CAPA) was distinctively lower than the creatinine-based eGFR (CKD-EPI). This was likewise true for no-CKD patients (Figure 2 left panel) and at-risk (TAVI) patients suffering from manifest kidney disease or bearing an increased risk (Figure 2 right panel). The cystatin C-derived eGFR (CAPA) was lower than the creatinine-derived eGFR (CKD-EPI) in ≈84% of no-CKD and ≈82% of at-risk (TAVI) cases.

In no-CKD patients, the CKD-EPI-calculated eGFR was 81.56 ± 12.41 mL/min/1.73 m^2^ in the mean and 83 mL/min/1.73 m^2^ in the median. In contrast, the eGFR derived from serum/plasma cystatin C (CAPA) was 66.38 ± 16.60 mL/min/1.73 m^2^ in the mean and 68 mL/min/1.73 m^2^ in the median. The prevalent downward bias of the cystatin C-based eGFR (CAPA) compared to the CKD-EPI-derived eGFR (−21.35 ± 18.64 and −13.50 ± 18.31% for males and females, respectively) was highly significant (*p* < 0.0001).

The at-risk (TAVI) patients exhibited lower eGFR values than the no-CKD patients. The CKD-EPI-derived eGFR was 66.14 ± 20.71 mL/min/1.73 m^2^ in the mean and 72 mL/min/1.73 m^2^ in the median. Nevertheless, the cystatin C-based eGFR (CAPA) was even lower (53.53 ± 16.92 mL/min/1.73 m^2^ in the mean and 53 mL/min/1.73 m^2^ in the median). Again, the downward bias of the cystatin C-based eGFR (CAPA) (−15.70 ± 19.16 and −18.09 ± 14.53% for males and females, resp.) was highly significant (*p* < 0.0001). An older formula that is also occasionally used is the MDRD formula [21], which is also based on serum creatinine. A similar result was also shown there, with a downward bias of cystatin C compared to this formula. In no-CKD patients, the MDRD-calculated eGFR was 83.82 ± 19.81 mL/min/1.73 m^2^ in the mean and 80.24 mL/min/1.73 m^2^ in the median. The prevalent downward bias of the cystatin C-based eGFR (CAPA) compared to MDRD (−24.38 ± 19.07 and −11.11 ± 19.85% for males and females, respectively) was highly significant (*p* < 0.0001). In the at-risk (TAVI) patient group, the MDRD-calculated eGFR was 70.81 ± 24.82 mL/min/1.73 m^2^ in the mean and 70.67 mL/min/1.73 m^2^ in the median. The prevalent downward bias of the cystatin C-based eGFR (CAPA) compared to the MDRD-calculated eGFR (−21.11 ± 18.54 and −21.78 ± 15.40% for males and females, respectively) was highly significant (*p* < 0.0001).

### 3.2. Potential Impact of eGFR (CAPA) on CKD Stage and Clinical Classification of Kidney Disease

**Figure 3:**
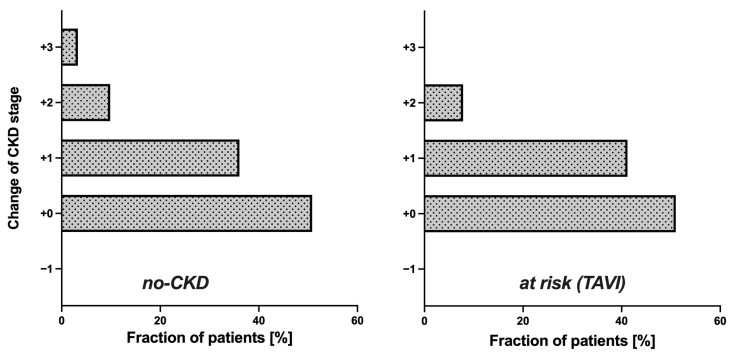
CKD restaging based on eGFR (CAPA): bars show percentage of patients. (**Left**): no CKD. (**Right**): at risk (TAVI).

**Figure 4:**
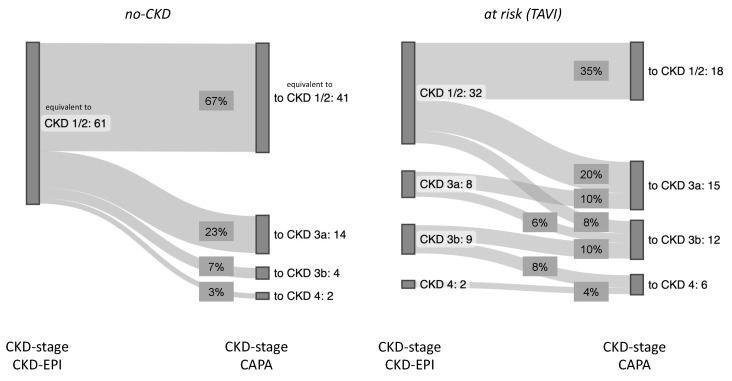
CKD-restaging based on eGFR CAPA: flows show proportions of patients per stage, restaging from CKD-EPI (**left**) to CAPA (**right**). Left diagram: no CKD. Right diagram: at risk (TAVI).

Since the cystatin C-based eGFR (CAPA) was notably lower than the eGFR (CKD-EPI) in both groups, the question arose whether the use of the eGFR (CAPA) instead of the eGFR (CKD-EPI) would result in assignments of the patients to different CKD stages. This question seemed particularly interesting for the no-CKD group, i.e., the patients classified as not suffering from kidney disease according to the eGFR (CKD-EPI). While half of these patients (50.82%) remained in the same CKD stage upon reclassification according to the eGFR (CAPA), the rest moved one (36.07%), two (9.84%), or even three (3.28%) CKD stages downwards (Figure 3 left). Also relevant, for one-third of these patients, the reclassification according to the eGFR (CAPA) resulted in a CKD stage indicative of kidney dysfunction or even kidney disease, while all these patients were deemed no-CKD according to the eGFR (CKD-EPI). Thus, the reclassification did not only alter the CKD stage but also the clinical classification of kidney disease state for a significant portion of the patients. A similar observation was made in the at-risk (TAVI) group: half of these cases (49.02%) were downgraded by one or two CKD stages (Figure 3 right), and 27.45% of the cases were moved to a worse clinical disease classification, i.e., from CKD stages 1/2 to CKD stages 3a/b or even 4 (Figure 4). It should be noted that not a single patient in both groups had an improved CKD stage or disease classification when based on the eGFR (CAPA) instead of the eGFR (CKD-EPI).

**Figure 5:**
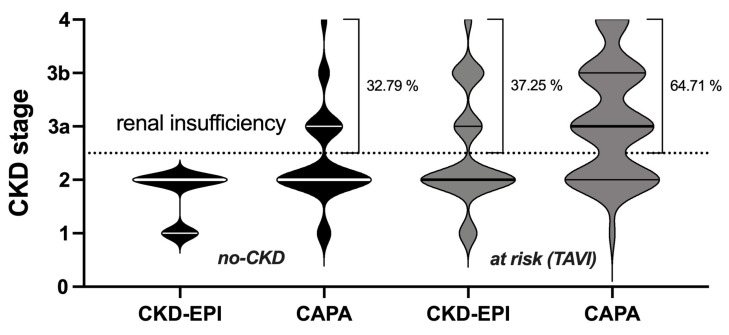
Distribution of CKD stages based on eGFR CKD-EPI and eGFR CAPA: CKD stages obtained for no-CKD (**left**) and TAVI-patients (**right**). Violins: proportion of patients. Thick lines: medians; thin lines: quartiles. Dotted line: border between renal sufficiency and insufficiency. Percentages: proportion of patients classified as renal insufficient.

Overall, the no-CKD patient cohort was entirely equivalent to CKD stage 1 or 2 according to the eGFR (CKD-EPI) and gained roughly one-third of cases of more or less advanced kidney insufficiency when reclassified according to the eGFR (CAPA). Similarly, in the at-risk (TAVI) group, roughly another one-third of the cases were judged to be at risk for chronic kidney failure when analyzed according to the eGFR (CAPA) (summarized in Figure 5).

### 3.3. Sensitivity of eGFR (CKD-EPI) and eGFR (CAPA) for Kidney-Relevant Clinical Diagnoses

To test whether the reclassification according to the eGFR (CAPA) from presumably no-CKD to presumed renal insufficiency (i.e., from CKD stage ≤ 2 to CKD stage > 2) was plausible in clinical terms, we unblinded the ICD-10-coded diagnoses of the (based on laboratory parameters) no-CKD group and identified etiologies plausibly encompassing or entailing kidney dysfunction or not. As summarized in Table 1, the vast majority (>90%) of those patients remaining in CKD stage 1 or 2 after recalculating the eGFR using cystatin C were indeed not diagnosed to suffer from primary or secondary renal disease. In contrast, a significant fraction (30%) of those patients who, according to the eGFR (CAPA), were moved from the no-CKD to the kidney-insufficient state (i.e., from CKD stage ≤ 2 to CKD stage > 2) had, indeed, diagnoses encompassing renal insufficiency.

### 3.4. Correlation of Serum Urea and eGFR

**Figure 6:**
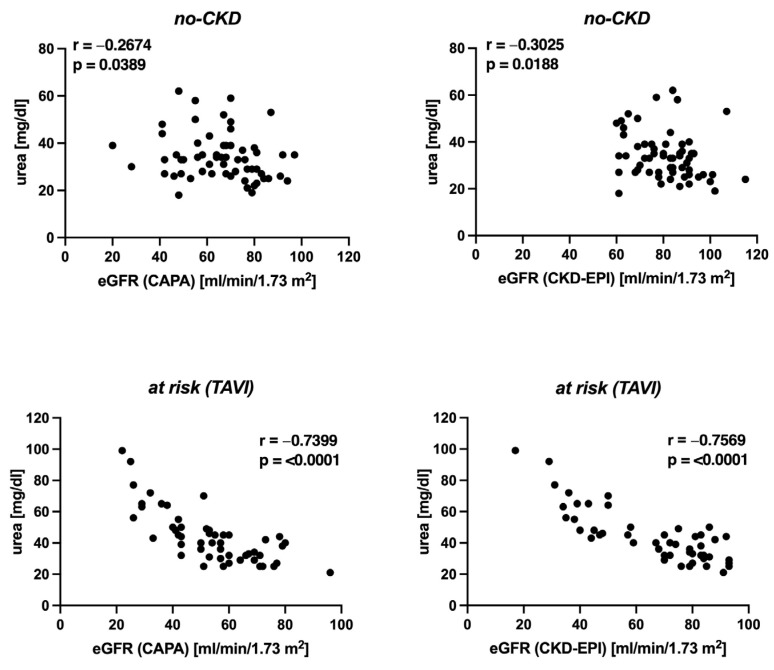
Spearman correlation between eGFR and serum urea: Results obtained for the no-CKD group (*n* = 60, **above**) and the at-risk (TAVI) group (*n* = 51, **below**). Each data point represents one patient. (**Left**): correlation between eGFR (CAPA) and serum urea. (**Right**): correlation between eGFR (CKD-EPI) and serum urea.

We also examined the correlation between eGFR and serum urea. The no-CKD group had a weak but significant correlation between serum urea and eGFR. This was true both when the eGFR was determined using cystatin C (CAPA) (r = −0.2674, *p* = 0.0389) and creatinine (CKD-EPI) (r = −0.3025, *p* = 0.0188) (Figure 6 above). In the at-risk (TAVI) group, there was a strong and significant correlation between serum urea and eGFR, both according to cystatin C (CAPA) (r = −0.7399, *p* = <0.0001) and creatinine (CKD-EPI) (r = −0.7569, *p* = <0.0001) (Figure 6 below).

## 4. Discussion

### 4.1. Sensitivity of First-Line Diagnostic for Kidney Dysfunction

Our data suggest that GFR estimations based on serum creatinine according to the CKD-EPI formula are prompting an overoptimistic clinical assessment of kidney function in multimorbid elderly persons (as typically hospitalized in a university hospital). This also applies to the GFR determination of patients with supposedly healthy kidneys. Consequently, unknown/hidden kidney disease is overlooked in up to 30% of cases with an undetermined state of kidney function. In the cohort of elderly hospitalized patients analyzed here, the GFR estimation based on cystatin C appeared more sensitive for manifest kidney insufficiency than creatinine-based GFR estimations. Our observation that the GFR is frequently underestimated by the CKD-EPI formula in elderly patients is plausible, given that the CKD-EPI formula has been developed based on a cohort encompassing relatively few individuals aged 60 years or more [21]. The observed downward bias of serum cystatin C-based relative to creatinine-based GFR estimates conforms to many previous findings [6,14,23].

### 4.2. Correct Assessment of CKD Stage

Our data obtained in a group of TAVI patients suggest that the severity of manifest kidney disease is often underestimated by GFR estimates based on serum creatinine according to the CKD-EPI formula. In the case of TAVI patients, the underdetermination of kidney insufficiency is particularly unfortunate because patients with renal dysfunction have a worse outcome after TAVI [4,5,6]. In addition to the current status assessment of renal function, the GFR determined by cystatin C in TAVI patients with a GFR of 3b or worse also seems to be better suited for the prognostic assessment of the patients. This is true for the risk of cardiovascular and cerebrovascular impairment [6].

Our data show that the proportion of these patients is higher than assumed according to standard diagnostics. Thus, correct pre-interventional assessments of renal function are essential for the post-interventional monitoring of these patients.

### 4.3. What Is the Ultimate Filtration Marker?

In agreement with previous studies [6,14,15,16,17], our data strongly suggest that cystatin C is superior to creatinine as a filtration marker. Nevertheless, cystatin C-based eGFR estimates can also be subject to error because the serum levels of cystatin C are confounded by extrarenal factors such as age, gender, body mass, body length, cigarette smoking, high low-density lipoprotein cholesterol, low high-density lipoprotein cholesterol, obesity, diabetes, and inflammation [24,25]. The clinical relevance of differences between eGFR values based on cystatin C and creatinine seems unclear. Thus, it has been shown that using the CKD-EPI cystatin C-based eGFR (in contrast to the CAPA formula employed here), especially to predict disease progression and mortality in patients with mild CKD, offers no benefits and only increases costs [26].

Given the limitations of both creatinine and cystatin C, other markers for measuring kidney function are currently the subject of research. For example, proenkephalin exhibited a more precise correlation with the GFR than creatinine in critically ill sepsis patients [27,28]. Unfortunately, proenkephalin has not yet been extensively compared to cystatin C. Thus, it remains to be studied whether proenkephalin is superior.

Our data indicate that serum urea correlates more strongly with the eGFR when the insufficiency is more severe. Therefore, it is not suitable for the first-line diagnostics of incipient kidney damage. In evaluating more advanced CKD, serum urea helps assess renal insufficiency. Elevated serum urea indicates a lower GFR. This is in line with the current standard diagnostics [11]. We can show that this correlation is not very different for the creatinine- or cystatin C-derived eGFR.

### 4.4. Limitations of Our Study

The limitations of our study are the small case number and the lack of GFR validation in the gold-standard GFR procedure. The height, weight, and the resulting BMI of patients were not considered as possible influencing factors. However, since many studies show that cystatin C appears superior to creatinine, it seemed sufficient to evaluate the additional value of cystatin C compared to creatinine in terms of clinical utility.

## 5. Conclusions

In the first-line diagnostics of elderly hospitalized patients for detecting previously unrecognized renal dysfunction, the sole use of creatinine as a GFR marker seems insufficient. Nevertheless, this procedure is still recommended in the German S2k interdisciplinary guidelines and the international KDIGO guidelines [8,29].

Furthermore, creatinine-based GFR estimation underestimates existing renal dysfunction compared to a cystatin C-based measurement.

Pending corroboration in a larger study, our results suggest modifying guidelines and recommendations concerning elderly hospitalized patients could be heralded. One could imagine that elderly hospitalized patients would significantly benefit from a cystatin C-derived determination of the eGFR (CAPA) as an obligatory diagnostic reflex whenever the creatinine-based eGFR (CKD-EPI) appears normal (i.e., is above 60 mL/min/1.73 m^2^).

## Figures and Tables

**Table 1:** Diagnoses related to CKD reclassification of the no-CKD group.

Reclassification (eGFR—CAPA)	N (%)	Etiologies ^1^ (Number of Patients)
Remained equivalent CKD stage 1/2	3 (7)	Renal disease: Acute or chronic kidney disease (1) Neoplasia (kidney) or loss of kidney (2)
	38 (93)	Nonrenal disease: Arthropathy (1) Autoimmune disease (2) Cardiovascular disease (1) Disease of the bile ducts (1) Disease of the eyes (2) Hematological disease (2) Infectious disease (1) Neoplasia/dysplasia (nonrenal) (7) Neurological disease (4) Rheumatic disease (13) Unknown/others (4)
Reclassified CKD stage 3a, 3b or 4	6 (30)	Renal disease: Acute or chronic kidney disease (3) Kidney transplant (1) Neoplasia (kidney) or loss of kidney (2)
	14 (70)	Nonrenal disease: Autoimmune disease (2) Disease of the eyes (1) Dysphagia (1) Flanks/abdominal pain (2) Gait and mobility disorders (1) Infectious disease (1) Neoplasia/dysplasia (nonrenal) (3) Rheumatic disease (3)

^1^ Derived from ICD-10-encoded diagnoses (Supplementary Table S1).

## Data Availability

The data presented in this study are available upon request from the corresponding author.

## Supplementary Materials

The following supporting information can be downloaded at: https://www.mdpi.com/article/10.3390/geriatrics8060120/s1, Table S1: Diagnoses linked to etiologies.

## Abbreviations

CAPA	Caucasian, Asian, pediatric, and adult
CKD	Chronic kidney disease
CKD-EPI	Chronic Kidney Disease Epidemiology Collaboration
CVD	Cardiovascular disease
eGFR	Estimated glomerular filtration rate
GFR	Glomerular filtration rate
ICD-10	International Statistical Classification of Diseases and Related Health Problems
IFFC	International Federation of Clinical Chemistry and Laboratory Medicine
KDIGO	Kidney Disease: Improving Global Outcomes
TAVI	Transcatheter aortic valve implantation
