# Supplementary material for: Questionable Validity of Creatinine-Based eGFR in Elderly Patients but Cystatin C Is Helpful in First-Line Diagnostics
Source: Geriatrics (Basel). 2023 Dec 8;8(6):120. doi: 10.3390/geriatrics8060120 (PMC10742602; doi:10.3390/geriatrics8060120)
Supplement: Supplementary file 1 [file geriatrics-08-00120-s001.zip › geriatrics-2639263-supplementary.pdf]

Supplementary Material: Table S1: Diagnoses linked to etiologies

etiology	Main or kidney-relevant secondary diagnosis (ICD-10-encoded)
Renal disease	
Acute or chronic kidney disease	*Chronic* kidney disease<br>*Acute* renal failure, Crush kidney with acute renal failure<br>*State* after acute renal failure
Kidney transplant	*Follow-up* examination after organ transplantation<br>*condition* after kidney transplantation
Neoplasia (kidney) or loss of the kidney	*Multilocular* cystic neoplasia of the kidney with low malignancy potential, status after renal cell carcinoma<br>*Loss* of kidneys, Follow-up care after living kidney donation<br>*Pulmonary* and osseous metastatic renal cell carcinoma<br>*Malignant* neoplasm of the kidney, angiomyolipoma at the upper pole of the right kidney
Non-renal disease	
Arthropathy	*Other* unspecified crystal arthropathies: shoulder region
Autoimmune disease	*Progressive* systemic sclerosis, other overlap syndromes, seronegative chronic polyarthritis: multiple localizations<br>*Other* giant cell arteritis<br>*MS* with a secondary chronic course with indication of an acute exacerbation or progression<br>*Other* forms of systemic lupus erythematosus
Cardiovascular disease	*Arterial* hypertension WHO grade II, no sec. cause of hypertension

Disease of the bile ducts	*Cholecystolithiasis*
Disease of the eyes	*Suspected* scleritis<br>*Zoster* ophthalmicus<br>*Corneal* dystrophy
Dysphagia	*Other* and unspecified dysphagia
Flanks/abdominal pain	*Pain* localized in other parts of the lower abdomen<br>*Other* and unspecified abdominal pain, Personal history of malignant neoplasms of other organs or systems, Hypothyroidism after medical interventions
Gait and mobility disorders	*Weakness*
Hematological disease	*Anemia*<br>*Status* after hematopetic stem cell transplantation, acute myeloid leukemia
Infectious disease	*Vesicular* stomatitis with enterovirus exanthema<br>*Infection* by corona viruses of unspecified location
Neoplasia/dysplasia (non-renal)	*Malignant* neoplasm of the rectum<br>*Prostate* dysplasia, Foreskin hypertrophy, phimosis and paraphimosis<br>*Prostate* adenocarcinoma<br>*Malignant* neoplasm of the prostate<br>*Malignant* neoplasm in the temporal lobe<br>*Malignant* neoplasm in the frontal lobe<br>*Olfactory* meningioma<br>*Malignant* neoplasm: urinary bladder, several parts overlapping<br>*Hepatocellular* carcinoma<br>*Locoregionally* recurrent, bipulmonary and lymphogenous metastatic p16-positive oropharyngeal carcinoma
Neurological diseases	

	Uninhibited neurogenic voiding, not elsewhere classified<br>*Cerebral* infarction due to embolism of cerebral arteries<br>*Gait* disturbance/ataxia
Rheumatic disease	*Other* seropositive chronic polyarthritis: Multiple localizations<br>*Other* seropositive chronic polyarthritis: Multiple localizations, systemic lupus erythematosus involving organs and organ systems<br>*Other* specified chronic polyarthritis: several localizations<br>*Seronegative* chronic polyarthritis: multiple localizations<br>*Felty* syndrome: Multiple localizations<br>*Psoriatic* arthropathy<br>*Polymyalgia* rheumatica<br>*Giant* cell arteritis in polymyalgia rheumatica, other polyarthrosis<br>*Wegener's* granulomatosis
Unknown/others	*Unknown*<br>*Follow-up* control with no evidence of neoplasia<br>*Preliminary* examination: living kidney donation
